# Morin-5′-Sulfonic Acid Sodium Salt (NaMSA) Attenuates Cyclophosphamide-Induced Histological Changes in Genitourinary Tract in Rats—Short Report

**DOI:** 10.3390/ph14030192

**Published:** 2021-02-26

**Authors:** Anna Merwid-Ląd, Dorota Ksiądzyna, Agnieszka Hałoń, Danuta Szkudlarek, Małgorzata Trocha, Marta Szandruk-Bender, Agnieszka Matuszewska, Beata Nowak, Tomasz Sozański, Anna Kuźniar, Adam Szeląg

**Affiliations:** 1 Department of Pharmacology, Wroclaw Medical University, ul. Mikulicza-Radeckiego 2, 50-345 Wrocław, Poland; dorota.ksiadzyna@umed.wroc.pl (D.K.); malgorzata.trocha@umed.wroc.pl (M.T.); marta.szandruk@umed.wroc.pl (M.S.-B.); agnieszka.matuszewska@umed.wroc.pl (A.M.); beata.nowak@umed.wroc.pl (B.N.); tomasz.sozanski@umed.wroc.pl (T.S.); adam.szelag@umed.wroc.pl (A.S.); 2 Department of Pathomorphology and Oncological Cytology, Wroclaw Medical University, ul. Borowska 213, 50-556 Wrocław, Poland; agnieszka.halon@umed.wroc.pl; 3 Department of Pathomorphology, Wroclaw Medical University, ul. Marcinkowkiego 1, 50-368 Wrocław, Poland; danuta.szkudlarek@umed.wroc.pl; 4 Department of Inorganic and Analytical Chemistry, Rzeszow University of Technology, al. Powstańców Warszawy 6, 35-959 Rzeszów, Poland; akuzniar@prz.edu.pl

**Keywords:** NaMSA, cyclophosphamide, histopathology, testis, urinary bladder

## Abstract

Cyclophosphamide (CPX) exerts toxicity in the urogenital system. The current study was designed to evaluate the effect of morin-5′-sulfonic acid sodium salt (NaMSA) on CPX-induced urogenital toxicity in rats. NaMSA (100 mg/kg/daily) and CPX (15 mg/kg/daily) alone or in combination and 0.9% NaCl (as a control) were given intragastrically for 10 days. Testes and epididymes from male and urinary bladders from male and female rats were evaluated histologically. In testes and epididymes, morphological changes and relative decrease in sperm count were assessed. In urinary bladders edema, hemorrhage and urothelium erosions were described by 0–2 points scoring system. Reproductive score (RS—in total 6 points) and urinary bladder score (BS—in total 6 points) were thereafter calculated. In CPX-receiving group RS (2.7) and BS (3.3) were significantly higher than in the control (0.5 and 0.25 for RS and BS, respectively). Co-administration of NaMSA reversed most of the morphological changes, which was reflected by lower RS and BS score (0.5 and 1.2 for RS and BS, respectively). The preliminary findings suggest that NaMSA may attenuate CPX-induced histological changes in rat urogenital tract.

## 1. Introduction

The male reproductive system is very sensitive to noxious factors and permanent or transient infertility is a great clinical problem, especially in younger patients. There are still not too many possibilities to counteract these drug-induced complications. Among many reasons of decreased fertility or even infertility such as anatomical pathologies, diabetes, obesity, smoking, cancers, radiation or environmental toxins, adverse effects of many anticancer agents are very important [1,2,3].

Cyclophosphamide (CPX) is a conventional anticancer drug belonging to the alkylating agents [4]. Despite introduction in clinical practice, many targeted treatment options as immunosuppressant or anticancer drugs [5], it is estimated that still every year many patients develop and are diagnosed with cancers, which are the indication for CPX administration [6].

Cyclophosphamide exerts significant adverse effects in the urinary tract and affects the male reproductive system, causing disorders of spermatogenesis with oligo- and azoospermia, testicular atrophy in men taking this drug and sometimes infertility, which may be irreversible. Lesions caused by CPX in the urinary tract are also well described and are observed after both short- or long-term treatment. In human hemorrhagic cystitis, pyelonephritis and hematuria are common complications [7]. With the mechanism of the urinary tract, especially the urinary bladder, injury is multifactorial and involves first of all the reaction of CPX metabolite (acrolein) with urothelium with subsequent inflammatory cascade. In the prevention of urinary toxicity, mainly life-threatening hemorrhagic cystitis, there are a few possibilities with good hydration, systemic administration of mesna (whose efficacy in this indication is recently discussed and doubtful), local irrigation of the urinary bladder with various substances or sometimes even surgical, invasive procedures [4,6]. Much less is known about the prevention of chronic injury of kidneys or the reproductive system. Therefore, there is still a great need to search for compounds that may decrease CPX-induced tissue toxicity instead or as adjuvant to standard prevention schedules and in this way, decrease mortality due to complications of anticancer therapy and increase patients’ quality of life. Plant-derived substances and their chemically modified derivatives are in the interest of scientists in a variety of medical indications. Many different compounds have been until now tested in experimental studies for reduction of cyclophosphamide-induced urogenital toxicity, especially in male rats using models of acute or chronic cyclophosphamide administration [8,9,10].

Morin-5′-sulfonic acid sodium salt (NaMSA) is a derivative of natural flavonoid morin, but as a sodium salt exerts much better water-solubility. We have described in our previous papers that NaMSA reversed some CPX-induced changes in oxidative stress parameters in liver and kidney in rat models of 10 days CPX administration at a daily dose of 15 mg/kg [11] and attenuated pathological changes in intestine morphology [12]. Generation of oxidative radicals may be one of the factors responsible for damages observed in male gonads and in the urinary tract [10,13,14,15]. In this paper, we describe preliminary results of the influence of NaMSA administration on CPX-induced toxicity in male rat gonads based on the histopathological evaluation of testes and epididymes and the effect of NaMSA on histological toxicity in the urinary bladders of both sexes.

## 2. Results

### 2.1. Tested and Epididymes Evaluation

**Figure 1:**
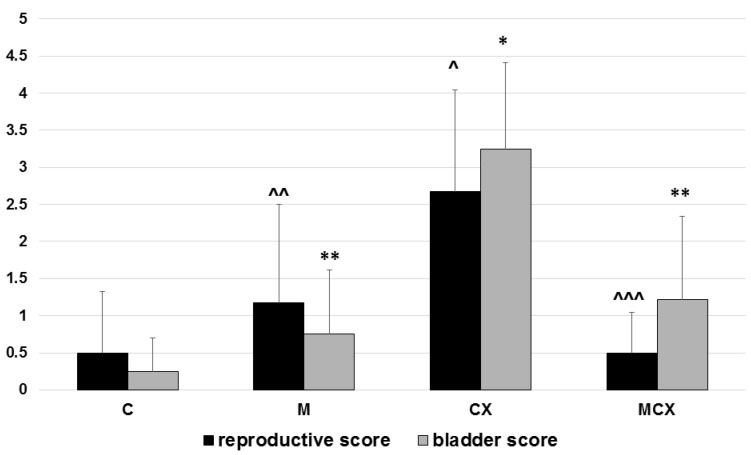
Total reproductive score (testes, epididymes and sperm, 0–6 points) and total urinary bladder score (hemorrhage, edema, erosions, 0–6 points). Scoring system described in the Material and Methods section. Bladder score: * *p* < 0.001, CX vs. C; ** *p* < 0.001, M vs.CX and MCX vs. CX. Reproductive score: ^ *p* < 0.01, CX vs. C; ^^ *p* < 0.05, M vs. CX and ^^^ *p* < 0.01, MCX vs. CX. Data presented as mean values and SD.

**Figure 2:**
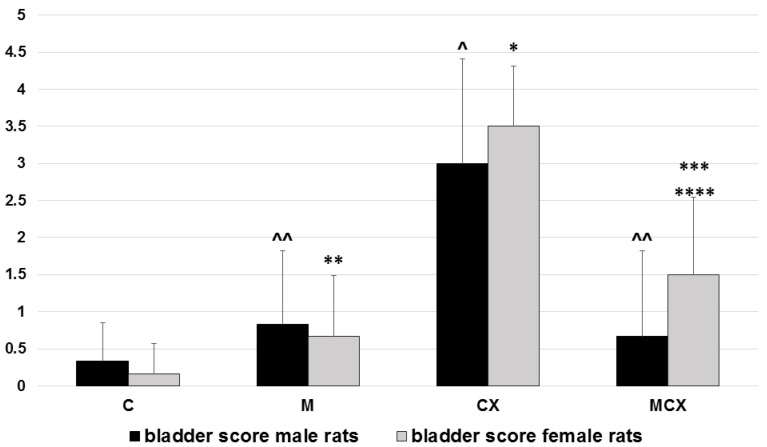
Total urinary bladder score (hemorrhage, edema, erosions, 0–6 points) in male and female rats. Scoring system described in the Material and Methods section. Male rats: ^ *p* < 0.001, CX vs. C; ^^ *p* < 0.01, M vs. CX and MCX vs. CX. female rats: * *p* < 0.001, CX vs. C; ** *p* < 0.001, M vs.CX; *** *p* < 0.01, MCX vs. CX; **** *p* < 0.05, MCX vs. C. Data presented as mean values.

**Figure 3:**
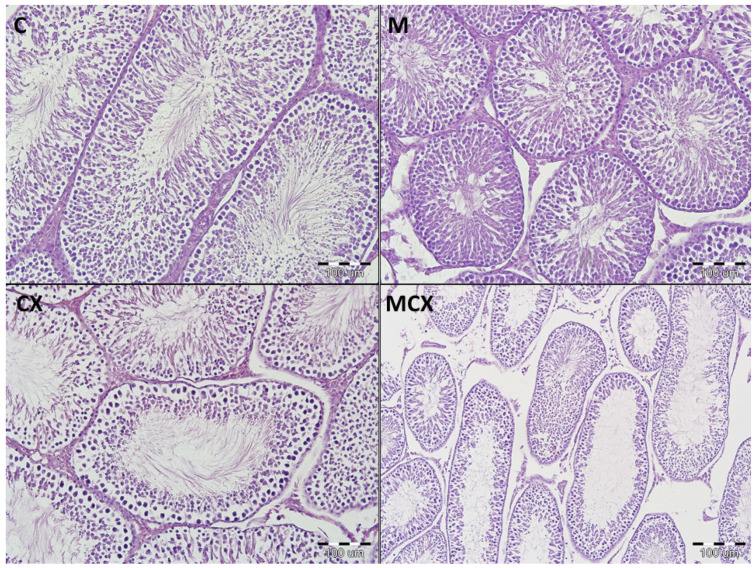
Histological cross-sections of testes (HE staining, magnification 200×). C—control group—appearance of normal testis with orderly maturation of germ cells from the base to the center of the lumen: numerous spermatogonia (along the basement membrane), primary and secondary spermatocytes, spermatids, and spermatozoa; M— Morin-5′-sulfonic acid sodium salt (NaMSA) receiving group—appearance of normal testis: the seminiferous tubules with numerous germ cells; CX—cyclophosphamide receiving group—changes grade 2—the seminiferous tubules with loss of germ cells, there is an orderly maturation of germ cells from the base to the center of the lumen, but the number of spermatogonia (along the basement membrane), primary and secondary spermatocytes, spermatids, and spermatozoa is decreased; MCX—group receiving cyclophosphamide and NaMSA—changes grade 1—the seminiferous tubules with discrete germ cells loss, there is an orderly maturation of germ cells from the base to the center of the lumen, but the number of spermatogonia (along the basement membrane), primary and secondary spermatocytes, spermatids, and spermatozoa is slightly reduced.

**Figure 4:**
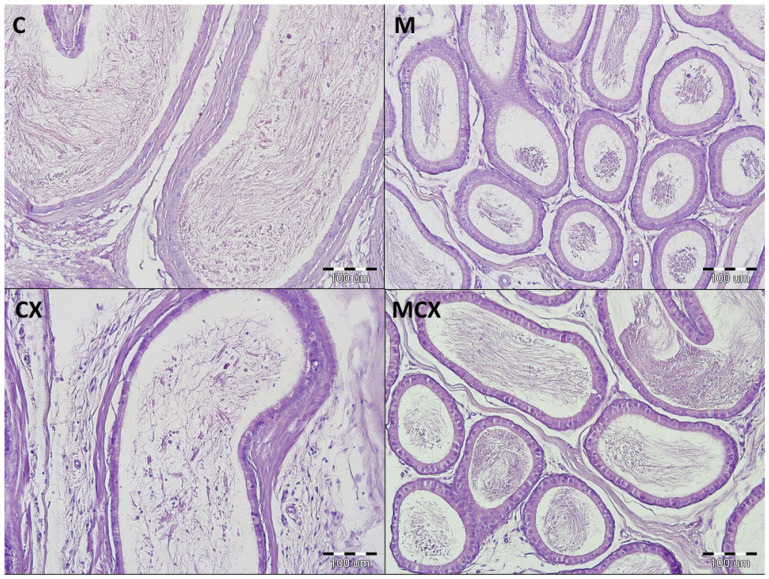
Histological cross-sections of epididymes (HE staining, magnification 200×). C—control group—no pathological changes, tubules have thick muscular coat, the lining is composed of tall, ciliated columnar cells, without atypia; M—NaMSA receiving group—no pathological changes, tubules have thick muscular coat, the lining is composed of tall, ciliated columnar cells, without atypia; CX—cyclophosphamide receiving group—changes grade 1—tubules have thick muscular coat, the lining is composed of tall, ciliated columnar cells, with some discrete changes; MCX—group receiving cyclophosphamide and NaMSA—no pathological changes, tubules have thick muscular coat, the lining is composed of tall, ciliated columnar cells, without atypia.

Results of the testes, epididymes and sperm score, and urinary bladder score are presented in Figure 1 and Figure 2. Intragastric CPX administration for 10 days in a daily dose of 15 mg/kg caused significant increase in testes and epididymes histological lesions described as irregular and diminished seminiferous tubules of a mild or severe grade along with various levels of decreased sperm count within the lumen of seminiferous tubules. Co-treatment with NaMSA in a daily dose of 100 mg/kg reversed those change, which was reflected by decreased testes and epididymes score. Representative histological changes for tested groups in testes and epididymes are presented in Figure 3 and Figure 4, respectively.

### 2.2. Urinary Bladder Evaluation

Hemorrhage, edema, and ulcerations/erosions in the urinary bladders were assessed and pathological changes were also the most significant in the CPX-receiving group. Similarly, treatment for 10 days with NaMSA reversed significantly CPX-induced changes reflected by decreased urinary bladder scoring system shown in Figure 1.

**Figure 5:**
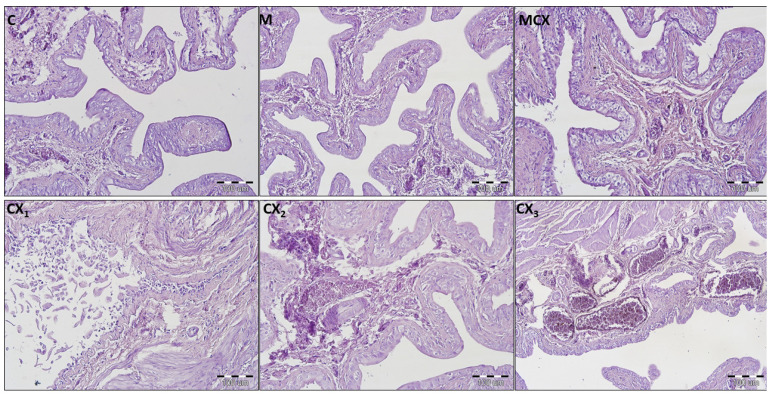
Histological cross-sections of urinary bladders (HE staining, magnification 200×). C—control group—no pathological changes; M—group receiving NaMSA—edema and discrete hyperemia of submucosa, there are normal 5–7 layers of transitional epithelium without atypia and some slightly dilated blood vessels; MCX—group receiving cyclophosphamide and NaMSA—edema of submucosa, there are normal 5–7 layers of transitional epithelium without atypia and some slightly dilated blood vessels; CX—group receiving cyclophosphamide—selected characteristic abnormalities; CX1—erosion of normal transitional epithelium without inflammation; CX2—edema and discrete hyperemia of submucosa, there are normal 5–7 layers of transitional epithelium without atypia and some slightly dilated blood vessels; CX3—high degree hyperemia of submucosa, there are normal 5–7 layers of transitional epithelium without atypia and some significantly dilated blood vessels.

Additionally, in urinary bladders, analysis of bladder score was performed separately for male and female rats and results as shown in Figure 2. In male rats, the most significant pathologies were found in the CPX-receiving group and the addition of NaMSA fully reversed these changes. In female rats, abnormalities caused by CPX were only partly reversed by NaMSA co-administration. Representative histological changes for tested groups for urinary bladders are presented in Figure 5.

## 3. Discussion

Cyclophosphamide belongs to the conventional anticancer agents but still is used as an important treatment option for many solid tumors or hematological malignancies, e.g., breast cancer or Hodgkin lymphoma. The drug is also a part of conditioning for a bone marrow transplantation or an alternative immunosuppressant in drug-resistant nephrotic syndrome in children or in systemic lupus erythematosus. Besides myelotoxicity typical for many anticancer drugs, cyclophosphamide causes significant adverse effects in the urinary tract and affects the male reproductive system with oligo- and azoospermia, testicular atrophy and sometimes irreversible infertility [7]. Hemorrhagic cystitis is a potentially life-threatening complication [16] and the recommended prevention with mesna administration with forced saline diuresis is still one of the most often chosen options, but not sufficient in all patients [17]. Therefore, there is a great need to search for new substances to increase efficacy of prevention of CPX-induced toxicities. Natural compounds, especially flavonoids, or their chemically modified derivatives are in the interest of many researches due to their potential anti-inflammatory and antioxidant activities. Morin-5’- sulfonic acid sodium salt (NaMSA) is a water-soluble derivative of naturally-occurred morin. Morin was found in many fruits and vegetables, e.g., *Psidium guajava*, *Acridocarpus orientalis*, *Moringa oleifera*, *Satureja hortensis*, all parts of almonds or in seaweed. Natural morin and NaMSA was found to scavenge free radicals and protect from damage caused by oxygen free radicals [18,19,20,21]. Among others, generation of free radicals is one of the postulated CPX-induced toxicity in various tissues [22]. We have previously found that NaMSA reversed CPX-induced decrease in superoxide dismutase activity and GSH level in rat kidneys [11] and protected from CPX-caused abnormalities in the gastrointestinal tract, revealed in a histological examination [12]. In this paper, we describe morphological changes in rat testes, epididymes, and urinary bladder after administration of CPX in a daily dose of 15 mg/kg for 10 days. CPX-induced changes in urogenital tract in animals are well described in various experimental models of CPX toxicity. The model of single intraperitoneal injection of CPX in a dose of 200 mg/kg is often used with the studied compound administered before or after the CPX injection to induce acute hemorrhagic cystitis or testicular toxicity [10,23,24], but other regimens of CPX administration are also applied, e.g., daily intraperitoneal injection of 100 mg/kg CPX dose for 3 days [25]. We chose 10 days intragastric administration of CPX in a low daily dose of 15 mg/kg with concomitant administration of NaMSA (100 mg/kg) as the potentially protective agent. This resembles more immunosuppressive rather than anticancer scheme, however, in our model we also found significant abnormalities in histological examination in testes, epididymes, and urinary bladders. In testes and epididymes, irregular and diminished seminiferous tubules were described as focal or diffuse. In urinary bladders edema, hemorrhage and urothelial erosions with desquamation of urothelium were found. Changes in the urinary bladders were less pronounced than described by other scientists in acute models of hemorrhagic cystitis induced by intraperitoneal injections of high-dose CPX. In this kind of CPX-induced bladder toxicity urothelial necrosis, vesical edema, increased urothelium thickness, erosion, ulceration, severe hemorrhage, inflammation, leukocyte infiltration, or even fibrosis were noticed [10,25,26].

Flavonoids were, and still are, an emerging topic for scientists due to many potential actions as free radicals scavenging, antitumor, or anti-inflammatory effects. Among various flavonoids, morin is of interest because it may heal injured cells [18]. Some environmental toxins such as titanium dioxide nanoparticles or mercuric chloride (HgCl2) upregulate expression of Bax and caspase-3 genes expression and downregulate Bcl-2. Morin or NaMSA may attenuate those changes which, next to the antioxidant activity of both substances, may protect from membrane damage resulting in decreased cell death and apoptosis [18,27].

Little is known about the protective effects of morin or its water-soluble derivative—morin-5′-sulfonic acid sodium salt—on damages caused by cyclophosphamide. Testicular toxicity is also an adverse effect of some other anticancer agents, e.g., procarbazine. Olaynka et al. [28] found that morin significantly restored the procarbazine-induced abnormal sperm parameters (count, motility, percentage of normal sperm). Some studies claim that morin protects testicular tissue from morphological injuries and pathology of sperm count and viability induced by titanium dioxide nanoparticles [27,29]. Pretreatment with morin significantly decreases expression of proinflammatory cytokines such as IL-1, TNF-alpha, NF-kappaB, and histological damage in rat kidney in doxorubicin-induced toxicity [30]. In vitro morin protects from apoptosis of renal proximal tubular cells (HK-2 cell line) caused by endoplasmic reticulum stress [31]. Recent studies also suggest that morin may increase anticancer activity of some conventional anticancer agents, e.g., fludarabine, inhibiting low molecular weight protein tyrosine phosphatase (LMW-PTP) [32], and may inhibit proliferation, migration, and invasion of EJ cells of bladder cancer via many different intracellular mechanisms [33].

To our best knowledge, little is known about the action of morin or its derivative such as NaMSA on damages caused by cyclophosphamide or other toxins in urinary bladder. Based on our preliminary findings, it may be suggested that NaMSA exerts some protective effects in the urinary bladder in rats receiving CPX in low daily doses. The exact mechanism of this activity requires further, detailed studies, also in the well-established acute model of CPX bladder toxicity. The NaMSA effect in urinary bladders seems to be more pronounced in male than female rats, but it also must be confirmed in the future. A study of Bon et al. [34] examined differences in CPX-induced bladder inflammation in male and female rats after administration of a single 100 mg/kg intraperitoneal dose. In general, they did not observe sex differences in bladder inflammation after CPX. Neither the time of day nor estrous cycle gave significantly different effects on the degree of cystitis but bladder inflammation in female rats was the most severe in rats in estrous stage when CPX was administered in the morning. Terado et al. [35] administered intraperitoneally CPX to ovariectomized or castrated rats and described typical histological changes in bladders in a form of submucosal edema, urothelial damage, hemorrhage, and leukocyte infiltration. Observed pathologies were more severe in ovariectomized females than in the CPX-administered sham-operated females, on the other hand, in male rats castration did not influence the severity of pathological changes in urinary bladder when compared to the sham-operated group. Treatment of ovariectomized females with estrogen ameliorated histological changes induced by CPX administration [35]. It was also found that estrogen administration decreases the severity of inflammation in the bladder in mice model of acute or chronic CPX-induced cystitis [36]. Early clinical data from very few series of cases also suggested some additional benefits in patients treated with conjugated estrogen who received CPX treatment, especially in decreasing hematuria. The mechanism of estrogen’s action in controlling the CPX-induced hematuria is still unclear, but some data suggest the action on cellular and cytokine immune response during inflammation, enhanced healing of damaged tissue, and/or improved endothelial cell function [37,38,39]. In our study, the females were not ovariectomized and we did not check the reproductive phase, so to make conclusions about differences in NaMSA action in bladders in male and females detailed studies are necessary. The degree of pathological changes in bladders of males and females, expressed in 0–6-point scoring system, was not significantly different.

## 4. Materials and Methods

### 4.1. Animals

An experiment was performed after the approval of the Local Ethics Committee for Animal Experiments in Wrocław at the Hirszfeld Institute of Immunology and Experimental Therapy of Polish Academy of Sciences and all experiments were performed in accordance with relevant named guidelines and regulations. There were 48 male and female Wistar rats, obtained from the Animal Research Center at Wroclaw Medical University (Wrocław, Poland), with average weight of 203.5 g ± 17.6 g, housed in standard laboratory conditions (12 h:12 h light-dark cycle, 21–23 °C, free access to standard rat chow and water) arranged in four experimental groups of 12 animals each (6 males and 6 females).

### 4.2. Design of the Experiment

The groups were as follows: C, M, CX, and MCX, receiving 0.9% saline solution only (Polpharma S.A., Starogard Gdański, Poland), NaMSA alone (100 mg/kg/day), cyclophosphamide alone (15 mg/kg/day, Sigma, Steinheim, Germany), and CPX (15 mg/kg/day) with NaMSA (100 mg/kg/day), respectively. CPX and NaMSA were administered intragastrically by the tube, after reconstitution in normal saline in 4 mL/kg volume for 10 days. Normal saline in group C was given in the equivalent volume. CPX was given at 9 a.m., whereas NaMSA was administered at 2 p.m. as it was described in earlier publications to minimize chemical interaction in the gastrointestinal tract [11,40,41]. Morin-5′-sulfonic acid sodium salt was synthesized at the Department of Inorganic and Analytical Chemistry, Rzeszow University of Technology, Poland, according to the methods described previously [42,43,44]. On the 11th day of the study rats were euthanized by dislocation of cervical vertebrae in deep peritoneal barbiturate anesthesia.

### 4.3. Histological Assessment

Testes and epididymes from male and urinary bladders from male and female rats were isolated, 4% buffered formalin fixed, paraffin-embedded and cut with a microtome into 5 μm thick slices and stained with hematoxylin-eosin for histological examination performed by two independent practiced two pathomorphologists. In testes and epididymes changes in spermatogenic epithelium and in seminiferous tubules epithelium were described as irregular and diminished seminiferous tubules with minimal, low grade (<25%) focal changes (1 point) or with moderate, high grade (≥25%) diffuse changes (2 points). Decreased amount of sperm within the lumen of seminiferous tubules was assessed according to the control group and described as minimal (1 point) or moderate (2 points). Every specimen was described by points (0–6), 0 points were given when no pathological changes were found in testes, epididymes and sperm count. Microscopic evaluation of urinary bladders assessed hemorrhage, edema, and urothelium loss. Each pathology was scored separately from 0 to 2 points. Scoring system of hemorrhage was as follows: 0 point—none, 1 point—mild, 2 points—severe and edema was scored as: 0 point—none, 1 point—flattening with submucosal edema, 2 points—severe. Erosions in urothelium were scored: 0 points—none, 1 point—minimal lesions, single cells loss, exfoliation of superficial epithelial cells, 2 points—significant mucosal erosion.

### 4.4. Statistical Analysis

Means ± standard deviation (SD) were used to describe experimental data. Statistical analysis of the effects of the drug on the studied parameters was performed using analysis of variance (ANOVA). After checking the equality of variances by Brown-Forsythe test, specific comparisons between studies groups were done with post-hoc NIR test. Hypotheses were considered positively verified if *p* < 0.05. Statistical analysis was performed using STATISTICA 13 PL (StatSoft, Kraków, Poland).

## 5. Conclusions

In conclusion, our preliminary findings suggest that NaMSA may prevent from morphological changes induced by CPX administration in the male reproductive system and in bladders of both sexes, especially in male rats, but this requires further research in other experimental models of CPX administration focused on detailed, potential mechanisms of such action.

## Data Availability

The data underlying this article will be shared on request to the corresponding author.
